# JTE-013 Alleviates Pulmonary Fibrosis by Affecting the RhoA/YAP Pathway and Mitochondrial Fusion/Fission

**DOI:** 10.3390/ph16101444

**Published:** 2023-10-12

**Authors:** Jiaxu Zhou, Yilan Song, Xingmei Wang, Xinrui Li, Chang Liu, Chenchen Tian, Chongyang Wang, Liangchang Li, Guanghai Yan, Hong Cui

**Affiliations:** 1 Jilin Key Laboratory for Immune and Targeting Research on Common Allergic Diseases, Yanbian University, Yanji 133002, China; zjx12335@163.com (J.Z.); songyl@ybu.edu.cn (Y.S.); 17765521183@163.com (X.W.); 2022010551@ybu.edu.cn (X.L.); Lc2294475795@163.com (C.L.); 15326338995@163.com (C.W.); lclee@ybu.edu.cn (L.L.); 2 Center of Medical Functional Experiment, Yanbian University Medical College, Yanji 133002, China; tcc18004305389@163.com; 3 Department of Anatomy, Histology and Embryology, Yanbian University Medical College, Yanji 133002, China

**Keywords:** JTE-013, S1PR2, lung epithelial cells, RHOA/YAP, mitochondrial fusion/fission, pulmonary fibrosis

## Abstract

Pulmonary fibrosis may be due to the proliferation of fibroblasts and the aggregation of extracellular matrix, resulting in the stimulation of inflammation damage, destroying lung tissue structure, seriously affecting the patient’s respiratory function, and even leading to death. We investigated the role and mechanism of JTE-013 in attenuating bleomycin (BLM)-induced pulmonary fibrosis. BLM-induced pulmonary fibrosis was established in mice. Type 2 alveolar epithelial cells (MLE-12) were stimulated with sphingosine monophosphate (S1P) in vitro. JTE-013, an S1PR2 (sphingosine 1-phosphate receptor 2) antagonist, and Verteporfin were administered in vivo and in vitro. IL-4, IL-5, TNF-α, and IFN-γ were measured by ELISA. IL-4 and IFN-γ positive cells were detected by flow cytometry. Inhibition of S1PR2 with JTE-013 significantly ameliorated BLM-induced pathological changes and inflammatory cytokine levels. JTE-013 also significantly reduced the expression of RHOA/YAP pathway proteins and mitochondrial fission protein Drp1, apoptosis, and the colocalization of α-SMA with YAP, Drp1, and Tom20, as detected by immunohistochemistry, immunofluorescence staining, TUNEL, and Western blot. In vitro, S1PR2 and YAP knockdown downregulated RHOA/YAP pathway protein expression, Drp1 phosphorylation, and Drp1 translocation, promoted YAP phosphorylation and phenotypic transformation of MFN2, and inhibited the up-regulation of mitochondrial membrane potential, reactive oxygen species production, and cell apoptosis (7.13% vs. 18.14%), protecting the integrity of the mitochondrial dynamics. JTE-013 also inhibited the expression of fibrosis markers α-SMA, MMP-9, and COL1A1, and alleviated the symptoms of pulmonary fibrosis. Conclusively, JTE-013 has great anti-pulmonary fibrosis potential by regulating RHOA/YAP and mitochondrial fusion/fission.

## 1. Introduction

Pulmonary fibrosis is a progressive lung disease characterized by the damage of alveolar epithelial cells, the activation of fibroblasts and myofibroblasts, the extensive deposition of extracellular matrix, excessive scarring, and eventually pulmonary necrosis [1]. Fibrosis is irreversible [2]. Pirfenidone and nintedanib are the approved anti-fibrosis drugs for pulmonary fibrosis. They can delay the progression of lung injury, but they cannot reverse or cure pulmonary fibrosis [3]. Thus, effective drugs for the treatment of pulmonary fibrosis are still needed.

Sphingosine-1-phosphate (S1P), is a bioactive sphingolipid mainly derived from red blood cells, platelets, and endothelial cells [4]. S1P can bind to S1P receptors (S1PRs) and is involved in the regulation of immune, cardiovascular, and neurological functions [5]. Many studies have confirmed that S1PR antagonists can effectively improve fibrosis [6,7]. JTE-013, a selective S1PR antagonist, can specifically inhibit the binding of S1P to Sphingosine 1-phosphate receptor 2/4 (S1PR2/4). JTE-013 is implicated in a variety of diseases, including asthma, liver injury, and cancer [8]. The main mechanism of S1P is to induce cell apoptosis, regulate cell survival and endothelial function, and affect vascular permeability and function of airway smooth muscle [9]. JTE-013 can suppress PI3K, Rho-associated kinase (ROCK), and PTEN, inhibiting vascular permeability and reducing the expression of NF-κB through CCL3 in the treatment of bronchial asthma [10]. In hepatocytes and cholangiocarcinoma cells, JTE-013 activates protein kinase B and extracellular signal-regulated kinase1/2 (ERK1/2) signaling pathways, thus attenuating S1P-induced cell migration and proliferation and treating liver injury in BDL mice [11]. JTE-013 can block NLRP3 inflammatory perfusion, inhibiting inflammation and the secretion of inflammatory cytokines IL-1β and IL-18, thus delaying liver inflammation and fibrosis [12]. JTE-013 can inhibit TGF-β1-induced cell migration and proliferation in A549 lung epithelial cells. The extracellular matrix accumulation and epithelial–mesenchymal transition lead to the directional movement of hepatic myofibroblasts to restore damaged tissues, mitigating the progress of liver fibrosis [13]. The use of JTE-013 in lung disease has mainly been observed in the study of asthma, wherein its effect on the inflammatory response has been significant. The regulatory effect of JTE-013 on epithelial cells and fibroblasts has also been observed in liver fibrosis [14,15]. Therefore, investigating the effects of JTE-013 on pulmonary fibrosis is necessary to determine its mechanism of action.

Yes-associated protein (YAP) and transcriptional coactivator with PDZ-binding motif (TAZ) are transcriptional co-regulators, which mainly bind to the enhancer elements by DNA binding platforms of TEAD factors [16]. RHOA and F-actin cytoskeleton are the primary determinants in the effects of Hippo kinases on YAP and TAZ, and the integrity of the F-actin cytoskeleton is crucial for large tumor suppressor gene 1 (Lats1) to inhibit YAP [17]. The YAP/TAZ is also involved in pulmonary fibrosis [18]. The RHOA/YAP pathway plays a role in tumor-stromal interactions [19]. The RHOA/YAP signaling pathway has been observed to increase ER stress levels, induce autophagy, and boost ROS expression, thereby promoting osteosarcoma cell metastasis. Inhibition of the RHOA/YAP pathway reduces the proliferation, invasion, and migration of malignant tumor cells in hepatocellular carcinoma [20,21]. RHOA/YAP inhibits fibroblast migration, invasion, proliferation, and collagen deposition, and suppresses the TGF-β/Smad pathway in vivo, thereby ameliorating fibrosis in proliferating vitreous [22]. The understanding of the RHOA/YAP signaling pathway is still limited, especially in fibrosis.

Mitochondria are essential organelles for cell metabolism, growth, and function. The homeostasis of mitochondria is regulated by autophagy, mitochondrial fusion, and fission [23], which is called mitochondrial dynamics. The major mitochondrial fusion proteins include mitofusin 1 (MFN1), mitofusin 2 (MFN2), and optic atrophy 1 (OPA1) [24]. The mitochondrial fission proteins include dynamin-related protein 1 (Drp1) and its mitochondrial receptor mitochondrial fission 1 (Fis1) [25]. Mitochondrial fission can generate new mitochondria and is important for the removal of damaged organelles by mitochondrial phagocytosis. The loss of mitochondrial fission can lead to fusion imbalance and an increase in the number of elongated mitochondria [24]. Subsequently, MFN1/2 expression and Drp1-mediated mitochondrial fragmentation are increased. The imbalance between mitochondrial fusion and fission processes changes the dynamics of reactive oxygen species (ROS), enhances ROS production and cell proliferation, leads to cell apoptosis and senescence, and promotes the progression of pulmonary fibrosis [26]. The potential of mitochondrial dynamics intervention in treating pulmonary fibrosis has been demonstrated [27]. DRP1-induced mitochondrial fission upregulates apoptosis regulators Bcl-2 and p53, which in turn promote myofibroblast apoptosis, and reduce fibroblast proliferation and collagen production, thus inhibiting lung parenchymal distortion and collagen deposition [28,29]. Inhibition of Drp-1-dependent mitochondrial fission not only prevents epithelial apoptosis, but also plays an important role in regulating fibrosis [30]. Knockdown of MFN2 inhibits mitochondrial autophagy and dysfunctional mitochondrial dynamics in macrophages, which in turn promotes renal fibrosis [31]. Therefore, regulating mitochondrial dynamics to attenuate mitochondrial division and promote mitochondrial fusion could be an effective target in treating fibrosis.

Mitochondrial defects are also significant contributors to the development of pulmonary fibrosis [32]. Impaired mitochondria and reduced expression of PTEN-induced putative kinase 1 are associated with mitochondrial dysfunction in AT2 cells of patients with idiopathic pulmonary fibrosis (IPF) [33]. Dysfunctional mitochondria, defective mitophagy, and impaired mitochondrial function contribute to cellular apoptosis and the pathogenesis of pulmonary fibrosis. Kobayashi et al. [34] observed a decrease in peroxisome proliferator-activated receptor gamma coactivator 1-alpha in IPF, which, in turn, amplified the signals associated with cellular contraction, matrix synthesis, fibroblast activation, and aging-related gene expression, leading to fibroblast pathological conditions. The regulation of myofibroblast differentiation and proliferation, along with the progression of pulmonary fibrosis, is influenced by mitochondrial autophagy and the reduction of the PARK2 gene, which mediates an autophagic defect and modulates the platelet-derived growth factor receptor/PI3K/AKT signaling pathway [35]. Matrix metalloproteinase-2 expression significantly increased, while caspase-3 activity decreased in the lung matrix of matrix metalloproteinase transgenic mice, thereby preventing apoptosis of alveolar epithelial cells and the development of pulmonary fibrosis [36]. Patients with IPF had a higher prevalence of common interstitial pneumonia compared to patients with familial IPF and genetic factors played a critical role in the risk of IPF onset [37].

In this study, we investigated the potential ameliorative effect of JTE-013, a receptor inhibitor of S1PR2, on pulmonary fibrosis. We selected the RHOA-mediated YAP pathway and the expression of growth factors that regulate downstream fibrosis-associated factors for analysis. It is worth noting that these specific mechanisms have not yet been extensively studied concerning pulmonary fibrosis. Additionally, we explored the effect of mitochondrial dynamics on fibrosis in epithelial cells, a concept that has not been previously proposed. Therefore, this paper provides an analysis and discussion of the mechanisms of the RHOA/YAP pathway and mitochondrial fusion/fission. Furthermore, we demonstrate for the first time that JTE-013 can regulate these two mechanisms to exert its effects. The experimental evidence presented in this study highlights the important role of JTE-013 in alleviating pulmonary fibrosis.

## 2. Results

### 2.1. JTE-013 Alleviates BLM-Induced Pathophysiological Changes in Mice with Pulmonary Fibrosis

**Figure 1:**
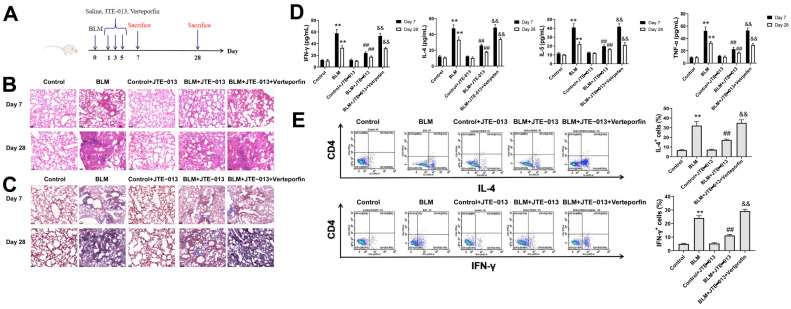
Effect of JTE-013 on pathological changes and levels of inflammatory factors in mice with pulmonary fibrosis. (**A**): Experimental procedure for pulmonary fibrosis model establishment in mice; (**B**): H&E staining of lung tissues (scale bar = 50 μm); (**C**): Masson staining of lung tissue (scale bar = 50 μm); (**D**): levels of IL-4, IL-5, IFN-γ, and TNF-α in BALF; (**E**): IL-4 and TNF-α positive cells in BALF (** *p* < 0.01 vs. control group, ## *p* < 0.01 vs. BLM group, && *p* < 0.01 vs. BLM + JTE-013 group).

The treatment procedure is shown in Figure 1A. The pathological changes of mouse lungs were analyzed using H&E (Figure 1B) and Masson staining (Figure 1C). The results showed that on day 7, there was inflammatory cell infiltration in the alveolar cavity and alveolar septum as well as obvious alveolar inflammation in the lung tissues of the BLM group. On day 28, the BLM group showed reduced inflammatory cell infiltration and fibrotic lesion formation, but increased deposition of mature collagen and aggravation of fibrosis. After the treatment with JTE-013, the infiltration of inflammatory cells and fibroblasts was reduced, and fibrosis was improved in the BLM + JTE-013 group. Verteporfin administration reversed the effects of JTE-013, as revealed by obvious pulmonary fibrosis in the lung tissues of the BLM + JTE-013 + Verteporfin group. ELISA was used to quantify the concentrations of inflammatory mediators IL-4, IL-5, IFN-γ, and TNF-α in the supernatants of mouse BALF (Figure 1D). Flow cytometry was used to quantify the proportions of IL-4 and IFN-γ positive cells in BALF (Figure 1E). After treatment with JTE-013, the expression levels of inflammatory cytokines IL-4, IL-5, IFN-γ, and TNF-α in BALF of the BLM + JTE-013 group were significantly decreased. Verteporfin administration reversed the effect of JTE-013. Thus, the JTE-013 intervention had the effect of ameliorating BLM-induced fibrosis in mice.

### 2.2. JTE-013 Inhibits the Expression of RHOA/YAP Pathway Proteins in Lung Tissues of Mice

**Figure 2:**
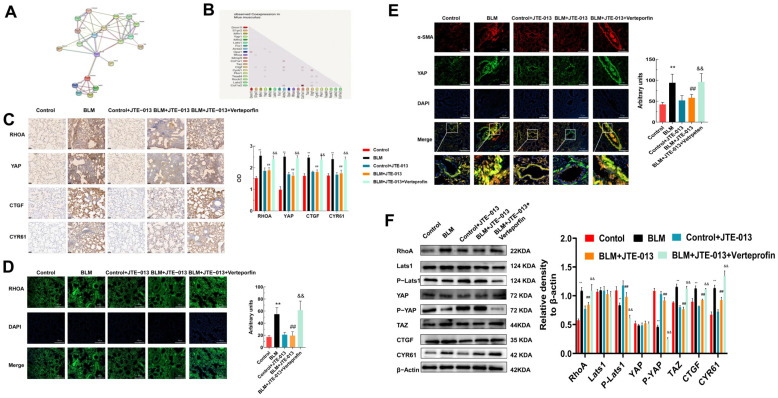
JTE-013 alleviated fibrosis by inhibiting the expression of RHOA, YAP, and related pathway proteins in lung tissues. (**A**): Protein–protein-interaction network constructed using the String database; (**B**): Co-expression of proteins retrieved from databases; (**C**): Immunohistochemical staining was used to detect the expression of RHOA, YAP, CTGF, and CYR61 and their OD quantitative analysis chart (scale bar = 50 μm). (**D**): Immunofluorescence staining was used to detect the expression of RHOA and their quantitative analysis chart (scale bar = 200 μm). (**E**): Immunofluorescence staining was used to detect the colocalization of YAP and α-SMA. (**F**): Western blot was used to detect the expression of RHOA, TAZ, CTGF, CYR61, and the phosphorylation of Lats1 and YAP. Their relative levels were analyzed and compared. (** *p* < 0.01 vs. control group, ## *p* < 0.01 vs. BLM group, && *p* < 0.01 vs. BLM + JTE-013 group). Original Western blot available in Supplementary Materials.

In this study, we investigated whether JTE-013 could regulate YAP through RHOA, thus mediating downstream proteins and mitochondrial dynamics. The protein–protein interaction network constructed by the STRING database is shown in Figure 2A. As shown in Figure 2B, YAP/TAZ and CTGF/CYR61 co-expressed with the fibrosis marker proteins and may have good anti-fibrotic activity. Immunohistochemical staining showed that the expression levels of RHOA, YAP, CTGF, and CYR61 decreased after treatment with JTE-013, and increased after further intervention with Verteporfin (Figure 2C). Immunofluorescence staining confirmed the change in RHOA expression (Figure 2D). As shown in Figure 2E, the colocalization of YAP and α-SMA was decreased after treatment with JTE-013 while it was increased after treatment with Verteporfin. Western blot revealed that JTE-013 treatment down-regulated the expression levels of RHOA, TAZ, CTGF, and CYR61, whereas it up-regulated the expression levels of p-YAP and p-Lats1 (Figure 2F). The changes in these proteins were reversed by Verteporfin. Thus, JTE-013 may exert an anti-fibrosis effect by affecting the RHOA/YAP pathway and CTGF/CYR61.

### 2.3. JTE-013 Restores the Expression of Mitochondrial Fusion/Fission and Fibrosis-Marker Proteins in Lung Tissues of Mice

**Figure 3:**
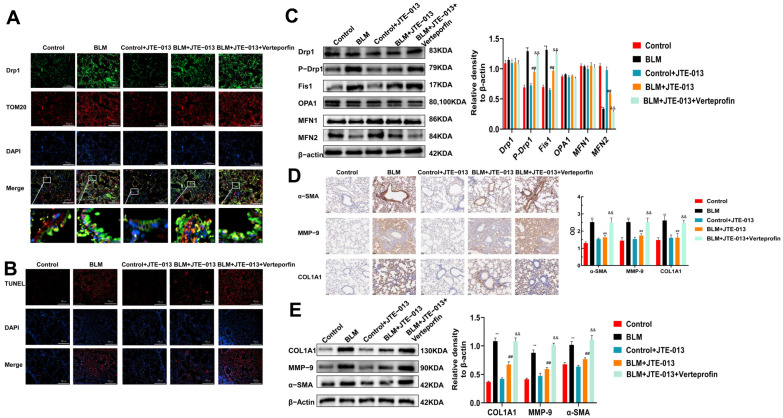
JTE-013 inhibited the expression of mitochondrial fusion/fission protein and fibrosis marker proteins in lung tissues. (**A**): Immunofluorescence staining was used to detect the colocalization of Drp1 and Tom20 (scale bar = 200 μm). (**B**): TUNEL staining was used to detect apoptosis in lung tissue. (**C**): Western blot was used to detect the expression of Fis1, OPA1, and MFN1/2 and the phosphorylation and transformation of Drp1. (**D**): Immunohistochemical staining was used to detect the expression of α-SMA, COL1A1, and MMP-9 and their OD quantitative analysis chart (scale bar = 50 μm). (**E**): Western blot was used to detect the expression of α-SMA, COL1A1, and MMP-9 (** *p* < 0.01 vs. control group, ## *p* < 0.01 vs. BLM group, && *p* < 0.01 vs. BLM + JTE-013 group). Original Western blot available in Supplementary Materials.

We then investigated the role of JTE-013 in mediating mitochondrial dynamics. Immunofluorescence staining showed that the colocalization of Drp1 and Tom20 in the lungs of the BLM group increased compared with the Control group (Figure 3A). Their colocalization decreased more in the BLM + JTE-013 group than in the BLM group, but increased more in the BLM + JTE-013 + Verteporfin group than in the BLM + JTE-013 group. TUNEL assay showed that the number of apoptotic cells in the lung tissue was decreased after treatment with JTE-013, while it was increased after Verteporfin treatment (Figure 3B). Western blot found that compared with the BLM group, the expression of Fis1 and phosphorylation of Drp1 in the lung tissues of mice in the BLM + JTE-013 group were significantly lower, but the expression of MFN2 was significantly higher (Figure 3C). These changes were reversed in the BLM + JTE-013 + Verteporfin group. However, the expressions of Drp1, MFN1, and OPA1 were not significantly changed. Immunohistochemical staining observed that JTE-013 treatment induced decreases in the fibrosis marker α-SMA, the extracellular matrix component COL1A1, and MMP-9, while their levels were increased by Verteporfin (Figure 3D). Western blot obtained similar results on the expression of α-SMA, COL1A1, and MMP-9 (Figure 3E). These data indicate that JTE-013 may affect the expression of mitochondrial fusion/fission and fibrosis-marker proteins, and Verteporfin reverses the effect of JTE-013.

### 2.4. JTE-013 Inhibits the Expression of RHOA/YAP Pathway and Fibrosis Marker Proteins in Alveolar Epithelial MLE-12 Cells

**Figure 4:**
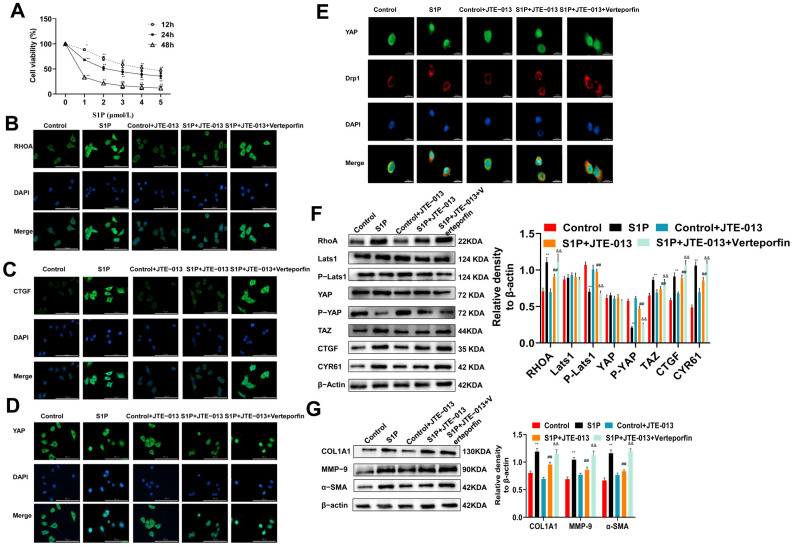
JTE-013 inhibited the expression of the RHOA/YAP pathway proteins and fibrosis marker proteins in alveolar epithelial MLE-12 cells. (**A**): MTT assay was used to detect the viability of MLE-12 cells treated with S1P * *p* < 0.05 vs. 0 μmol/L, ** *p* < 0.01 vs. 0 μmol/L). (**B**–**D**): Immunofluorescence staining was used to detect the expression of RHOA (**B**), CTGF (**C**), and YAP (**D**) (scale bar = 100 μm). (**E**): Immunofluorescence staining was used to detect the colocalization of YAP and Drp1 (scale bar = 20 μm). (**F**): Western blot was used to detect the expression of RHOA, TAZ, CTGF, CYR61, and the phosphorylation of Lats1 and YAP. (**G**): The expressions of α-SMA, COL1A1, and MMP-9 were detected by Western blot. (** *p* < 0.01 vs. control group, ## *p* < 0.01 vs. S1P group, && *p* < 0.01 vs. S1P + JTE-013 group). Original Western blot available in Supplementary Materials.

S1P was used to directly stimulate MLE-12 cells and then the effect of JTE-013 on the fibrosis of MLE-12 cells was investigated. MTT assay observed that with the increase in S1P concentration, the viability of cells decreased gradually (Figure 4A). Finally, the treatment with 3 μmol/L S1P for 24 h was determined as the optimal condition for subsequent experiments. Immunofluorescence staining showed that the expression levels of RHOA and CTGF protein were decreased by JTE-013, but increased by Verteporfin (Figure 4B,C). The YAP in the S1P group was translocated from the cytoplasm to the nucleus. After treatment with JTE-013, the expression of YAP in the nuclear was weakened, and part of it was translocated from the nucleus to the cytoplasm (Figure 4D). Verteporfin treatment restored the expression and localization of the YAP protein. The co-localization of YAP and Drp1 decreased after treatment with JTE-013, and increased after Verteporfin intervention (Figure 4E), demonstrating the relationship between YAP and Drp1, and deepening the understanding of the relationship between RHOA/YAP pathway and mitochondrial dynamics. Western blot analysis of RHOA, TAZ, CTGF, CYR61, YAP, p-YAP, Lats1, and p-Lats1 expressions in MLE-12 cells showed similar results to those in lung tissues, confirming the consistency between in vitro and in vivo results (Figure 4F). Additionally, the expression changes of α-SMA, COL1A1, and MMP-9 proteins in MLE-12 cells were also similar to those in the lung tissue samples (Figure 4G). Therefore, the anti-fibrotic effect of JTE-013 via the RHOA/YAP pathway was confirmed in vitro.

### 2.5. Regulation of S1PR2 Knockdown on Mitochondrial Fusion/Fission and Mitochondrial Membrane Potential in MLE-12 Cells

**Figure 5:**
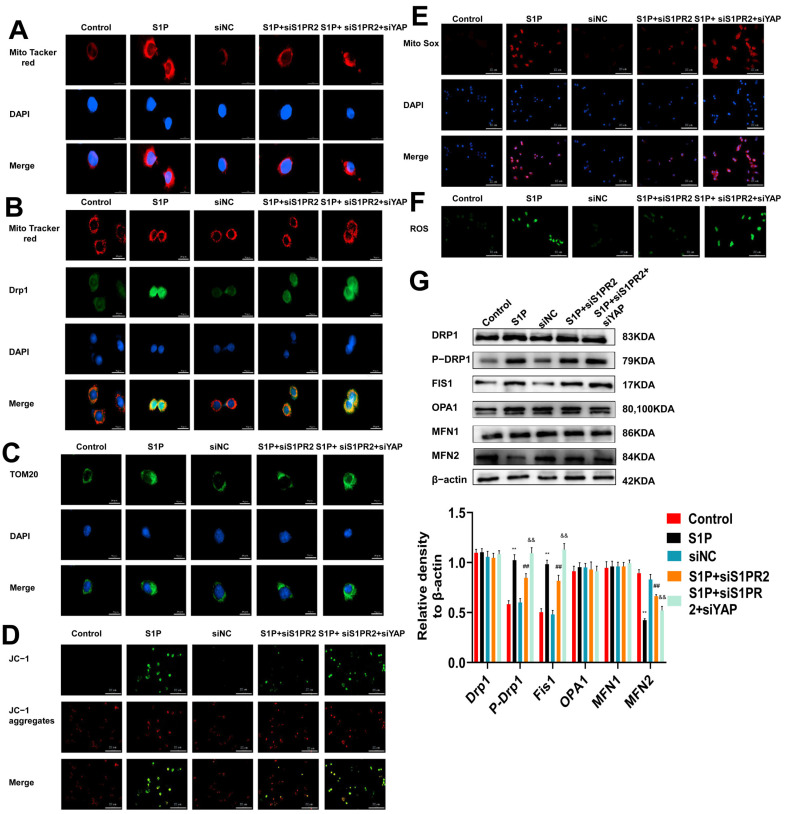
Regulation of mitochondrial fusion/fission by S1PR2 siRNA in MLE-12 cells. (**A**): Mito tracker red immunofluorescence staining was used to observe the changes in mitochondria (scale bar = 20 μm). (**B**): Colocalization of mitochondria and Drp1 was detected by immunofluorescence staining. (**C**): Immunofluorescence staining was used to detect the expression of Tom20. (**D**): JC-1 immunofluorescence staining was used to detect the level of mitochondrial membrane potential (The scale bar = 200 μm). (**E**): The production of mitochondrial ROS was detected by immunofluorescence staining of Mito SOX. (**F**): The production of ROS was detected by immunofluorescence staining of DCFH-DA. (**G**): Western blot was used to detect the expression of Fis1, OPA1, and MFN1/2, and the phosphorylation of Drp1. (** *p* < 0.01 vs. control group, ## *p* < 0.01 vs. S1P group, && *p* < 0.01 vs. S1P + siS1PR2 group). Original Western blot available in Supplementary Materials.

To further explore the effects of S1PR2 and YAP on mitochondrial dynamics, we knocked down the expressions of S1PR2 and YAP using siRNA. The Mito tracker red and colocalization of mitochondria with Drp1 in MLE-12 cells were detected by immunofluorescence staining. As shown in Figure 5A,B, the mitochondrial fission and the colocalization of mitochondrial and Drp1 decreased after knocking down S1PR2. The knockdown of S1PR2 and YAP recovered the levels of mitochondrial fission, Drp1 expression, and the colocalization of mitochondrial and Drp1. The immunofluorescence staining of Tom20 showed similar results as Drp1 (Figure 5C). JC-1 staining showed that the mitochondrial membrane potential returned to the normal level after knocking down S1PR2, while when knocking down both S1PR2 and YAP, the membrane potential decreased again (Figure 5D). The production of mitochondrial ROS (Figure 5E) and ROS (Figure 5F) was decreased after the S1PR2 knockdown, while their production returned to a higher level after the S1PR2 knockdown and YAP knockdown. Western blot found that the changes of Drp1, p-DRp1, Fs1, OPA1, MFN1, and MFN2 in MLE-12 cells were similar to those in lung tissues. These results confirm that siS1PR2 and siYAP may mediate the phenotypic transformation of fusion protein MFN and the phosphorylation of fission protein Drp1, affecting the changes of mitochondrial membrane potential, the production of mitochondrial ROS and ROS, and mitochondrial dynamics in MLE-12 cells.

### 2.6. Regulation of JTE-013 on Mitochondrial Fusion/Fission and Mitochondrial Membrane Potential in MLE-12 Cells

**Figure 6:**
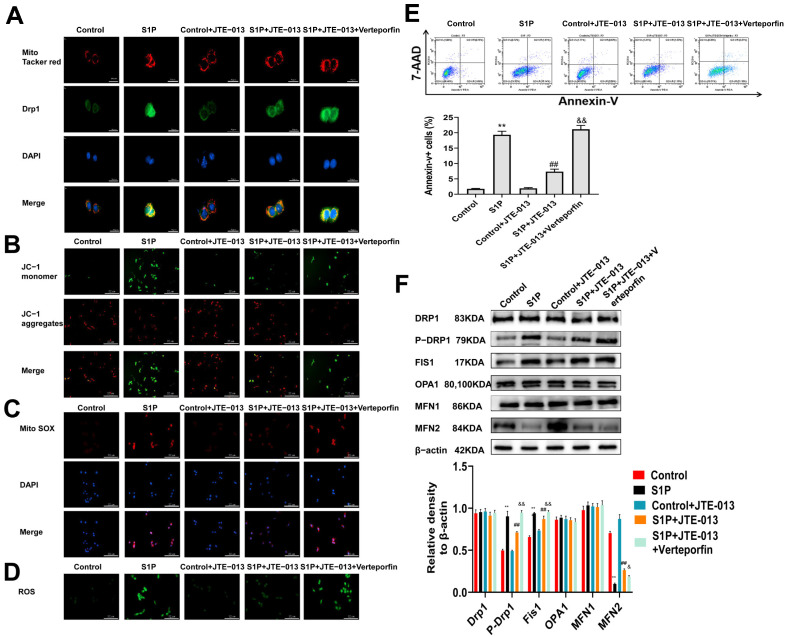
Regulation of mitochondrial fusion/fission by JTE-013 in MLE-12 cells. (**A**): Colocalization of mitochondria and Drp1 was detected by immunofluorescence staining (scale bar = 20 μm). (**B**): The mitochondrial membrane potential was detected by JC-1 immunofluorescence staining (scale bar = 200 μm). (**C**): The level of mitochondrial ROS was detected by immunofluorescence staining of Mito SOX. (**D**): DCFH-DA immunofluorescence staining was used to detect the level of ROS. (**E**): The apoptosis of MLE-12 cells was analyzed by flow cytometry. (**F**): Western blot was used to detect the expression of Fis1, OPA1, and MFN1/2, and the phosphorylation and transformation of Drp1. (** *p* < 0.01 vs. control group, ## *p* < 0.01 vs. BLM group, & *p* < 0.05 vs. BLM + JTE-013 group, && *p* < 0.01 vs. BLM + JTE-013 group). Original Western blot available in Supplementary Materials.

We further assessed the effect of JTE-013 on mitochondrial fusion/fission and mitochondrial membrane potential. Immunofluorescence staining was used to detect the colocalization of mitochondria and Drp1 (Figure 6A), mitochondrial membrane potential (Figure 6B), mitochondrial ROS (Figure 6C), and ROS (Figure 6D) in MLE-12 cells, and the results were similar to those of S1PR2 knockdown. This confirms that JTE-013 exerts a similar effect to S1PR2 knockdown. Flow cytometry was used to determine whether the effect of S1P on MLE-12 cell viability is mediated by apoptosis. The results showed that the proportion of apoptotic cells increased in the S1P group, which was reduced by the JTE-013 treatment but increased by the Verteporfin treatment (Figure 6E). Additionally, JTE-013 treatment induced similar changes to S1PR2 knockdown in the levels of Drp1, p-DRp1, Fs1, OPA1, MFN1, and MFN2 proteins (Figure 6F). Hence, the effect of JTE-013 on mitochondrial fusion/fission was similar to that of the S1PR2 knockdown.

## 3. Discussion

In recent years, there has been a marked increase in the incidence of respiratory diseases [38]. The main cause of chronic respiratory disease is persistent inflammation [39]. Identifying promising targeted therapeutic factors is crucial for addressing this issue. S1P, which is a key component of sphingolipid metabolism and acts primarily on downstream S1PRs, has been shown to respond to acute stimuli and plays an important role in advanced respiratory disease [38,40,41]. Moreover, JTE-013 and S1PR2 knockdown significantly reduced the proliferation of cholangiocytes, cholestatic injury, inflammation, and liver fibrosis by acting on ERK1/protein kinase B [11]. Meanwhile, JTE-013 can reduce the secretion and activation of NLRP3 and IL-1β through the S1PR2/p38 MAPK/YAP pathway as well as significantly reduce the pathological damage of liver tissue, the accumulation of collagen, liver injury, and fibrosis [42,43]. Chiba Y et al. found that S1PR2 and RHO/ROCK signaling reduced the contraction of bronchial smooth muscle and alleviated asthma [44]. JTE-013 can improve erectile dysfunction by inhibiting the RHO/ROCK kinase pathway and fibrosis [45]. Since S1P plays a pro-inflammatory role in most diseases, S1PR2 antagonism with JTE-013 can reduce the inflammation of the disease to exert alleviating effects [46]. S1PR2 has also been widely reported to affect YAP, reduce the invasive growth of cells, and promote the differentiation of AT2 into AT1 required for alveolar repair [47,48]. In this study, we demonstrated the potential of JTE-013 to regulate the RHOA/YAP pathway and mitochondrial dynamics in the context of pulmonary fibrosis. Through both in vitro and in vivo experiments, we observed that JTE-013 effectively reduced the expression of fibrosis-related factors including MMP-9, COL1A1, α-SMA, as well as growth factors CTGF and CYR61. Furthermore, JTE-013 demonstrated efficacy in improving mitochondrial dysfunction, a consequence of pulmonary fibrosis, primarily by modulating the expression of mitochondrial fission protein Drp1 and fusion protein MFN, reducing oxidative stress levels, and enhancing apoptosis. These findings provide valuable insights into the pathophysiology of pulmonary fibrosis and offer potential avenues for further research in the field of respiratory diseases.

In the present study, we investigated the potential effects of JTE-013 on BLM-induced lung tissue fibrosis in mice as well as the RHOA/YAP pathway and mitochondrial fusion/fission changes induced by S1P in MLE-12 cells. JTE-013 was found to regulate the physiological processes of pulmonary fibrosis and reduce BLM-induced inflammatory pulmonary fibrosis, as evidenced by the results of H&E and Masson staining, and, the expression changes of IL-4, IL-5, IFN-γ, and TNF-α in BALF. In addition, JTE-013 reduced the proportion of IL-4 and TNF-α positive cells in BALF and spleen. Studies have shown that JTE-013 participates in the acute immune response, reduces the levels of histamine, inflammatory factors IL-4, IL-13, IL-17, and IFN-γ in mice, reduces the maturation and migration of dendritic cells, and participates in the degranulation of mast cells, which is involved in dermatitis [49] and asthma [15,40].

We also found that JTE-013 reduced the expression of RHOA, TAZ, CTGF, and CYR61, and increased the phosphorylation of YAP and Lats1 in the RHOA/YAP pathway. JTE-013 also reduced the colocalization of YAP with Drp1 and α-SMA. Furthermore, JTE-013 significantly reduced the expression of fibrosis markers MMP-9, COL1A1, and α-SMA. Verteporfin intervention reversed most of the effects of JTE-013, suggesting that JTE-013 exerts its effects by regulating YAP. YAP can reduce mitochondrial oxidative damage in cardiomyocytes [50]. Drp1 and YAP, which can regulate mitochondria through SIRT1/MFN2, can reduce gastric cancer survival and migration [51], and attenuate mitochondrial dysfunction in cardiac hypertrophy [52]. S1PR2 antagonist can ameliorate high-glucose-induced mitochondrial fission and dysfunction, increase ROS production, and reduce NO levels by regulating RHO/ROCK [53]. It can also reduce ROS levels through IL-8 and TNF-α [54]. In our study, JTE-013 attenuated Drp1-TOM20 colocalization, decreased apoptosis levels, Drp1 phosphorylation, and Fis1 expression, promoted MFN1/2 transformation expression, and alleviated mitochondrial membrane potential changes, ultimately reducing the production of ROS and mitochondrial ROS production and Drp1 translocation. A study suggested that MFN2, but not MFN1 and OPA1, was attenuated in the cells primarily after inflammatory injury [55]. These results suggest that JTE-013 has the potential to alleviate fibrosis and improve mitochondrial dysfunction through RHOA/YAP. S1P has also been shown to affect mitochondrial autophagy and can regulate the formation of the autophagy signaling network through the S1PR2/S1PR3 axis, thus facilitating liver transplantation [56]. S1PR2 has also been shown to regulate autophagy in the lung [57]. Therefore, S1PR2 may improve pulmonary fibrosis and be involved in the mitochondrial biological processes by regulating YAP.

## 4. Materials and Methods

### 4.1. Reagents

Bleomycin (BLM) (#203401), S1P (#73914), and JTE-013 (#SML0700) were purchased from Sigma-Aldrich (St. Louis, MO, USA). Verteporfin (YAP Inhibitor) (#CL318952) was from MCE (Turlock, CA, USA). Mouse Interleukin-4 (IL-4) ELISA Kit (#ELM-IL4), Mouse Interleukin -5 (IL-5) ELISA Kit (#ELM-IL5), and Mouse interferon-γ (IFN-γ) ELISA Kit (#ELM-IFN-γ) were from Ray Biotech Inc. (Wuhan, China). Mouse Tumor Necrosis Factor-α (TNF-α) ELISA Kit (#BMS607-3) and the intracellular fixation and permeabilization kit (#88-8824-00) were provided by Invitrogen (Shanghai, China). Hematoxylin–eosin (H&E) staining (#G1120), Masson staining (#G1345), and BCA Protein Assay Kit (#PC0020) were from Solarbio (Beijing, China). DMEM/F12 (#01-172-1A) and fetal bovine serum (#04-001-1ACS) were from the Biological Industry (Beihai, China). The antibodies of rabbit anti-alpha smooth muscle actin (α-SMA) (#19245), rabbit anti-β-actin (#4970), rabbit anti-MFN2 (#9482S), rabbit anti-OPA1 (#80471S), rabbit anti-OPA1 (#367589), rabbit anti-P-Drp1 (#3455S), rabbit anti-mitochondrial outer membrane receptor (Tom20) (#42406), mouse anti-Drp1(#14647), anti-mouse IgG (H + L), F (ab’) 2 Fragment (Alexa Fluor^®^ 488 Conjugate) (#4408), and anti-rabbit IgG (H + L), F (ab’) 2 Fragment (Alexa Fluor^®^ 488 Conjugate) (#4412) were from Cell Signaling Technology (Boston, MA, USA). Rabbit anti-collagen, type I alpha 1 (COL1A1) (#ab34710), rabbit anti-matrix metalloprotein-9 (MMP-9) (#ab38898), rabbit anti-Drp1 (#ab184247), rabbit anti-Fis1 (#ab229969), and goat anti-mouse IgG H&L (Alexa Fluor^®^ 568) (#ab145473) were from Abcam (Boston, MA, USA). Rabbit anti-MFN1 (#DF7543) was obtained from Affinity (San Antonio, TX, USA). Rabbit anti-RHOA (#bs-1180R), rabbit anti-Lats1 (#bs-2904R), rabbit anti-P-Lats1 (#bs-3246R), rabbit anti-TAZ (#bs-12367R), rabbit anti-connective tissue growth factor (CTGF) (#bs-0743R), Rabbit anti-Human Cysteine Rich Protein, Angiogenic Inducer 61 (CYR61) (#bs-1290R) were purchased from Bioss (Beijing, China). Rabbit anti-YAP (#BS9920M), and Rabbit anti-P-YAP (#BS65509) were from Bioworlde (Bloomington, IN, USA). MTT (#NC-S0006-01) and MitoTracker Red (#M7521) were from Life Science (Westminster, CO, USA). DNase-free proteinase K (#ST532), Dead End Fluorometric TUNEL System (#C1089), RIPA (#P0013B), JC-1 (#C2006), BCA assay kit (#P0010S), and DCFH-DA (#S0033S) were from Beyotime (Shanghai, China). Lipofectamine RNAiMAX reagent (#13778030) and MitoSOX (#M36008) were provided by Thermo Fisher Scientific (Waltham, MA, USA). FITC Annexin V Apoptosis Detection Kit (#559763), APC Rat Anti-Mouse IL-4 (#554436), and APC Rat Anti-Mouse IFN-γ (#554413) were from BD Biosciences (SanJose, CA, USA). PE IL-5 Monoclonal Antibody (#12-7052-82) was from eBioscience ((San Diego, CA, USA). APC Anti-mouse TNF-α (#506308) was from Biolegend (San Diego, CA, USA).

### 4.2. Animals

Fifty male BALB/c mice were obtained from the Experimental Animal Center of Yanbian University. They were kept in standard conditions. All animal experiments were approved by the Animal Protection and Use Committee of Yanbian University (No: YB. No20210620b060; Date: 2018-05-22).

### 4.3. Animal Treatment and Grouping

After 1 week of acclimation, mice were randomly divided into the Control group, BLM model group, JTE-013 group, BLM + JTE-013 group, and BLM + JTE-013 + Verteporfin group (10 mice in each group). Mice in the Control group and JTE-013 group were intratracheally injected with normal saline (100 μL) on day 0 and were intraperitoneally injected with normal saline (100 μL) and JTE-013 (8 mg·kg^−1^, 100 μL) on day 1, 3 and 5, respectively. The BLM, BLM + JTE-013, and BLM + JTE-013 + Verteporfin groups were intratracheally injected with BLM (10 mg·kg^−1^, 100 μL) on day 0, and intraperitoneally injected with normal saline (100 μL), JTE-013 (8 mg·kg^−1^, 100 μL), and JTE-013 (8 mg·kg^−1^, 100 μL) and Verteporfin (10 mg·kg^−1^, 100 μL) on day 1, 3, and 5, respectively.

### 4.4. Sample Collection

On days 7 and 28 after JTE-013 and Verteporfin administration, mice (*n* = 5 in each group) were sacrificed and sample collection was performed. The lungs were lavaged with sterile saline to collect BALF (bronchoalveolar lavage fluid). After centrifugation, the supernatant was subjected to cytokine detection. The expression of IL-4, IL-5, TNF-α, and IFN-γ in BALF was analyzed by ELISA and flow cytometry. Subsequently, the left and right lungs and spleen were collected.

### 4.5. Histological Analysis

Lung tissues were fixed in 4% paraformaldehyde. After the routine procedures of dehydration and embedding, the lung sections were subjected to H&E and Masson staining. The inflammatory cell infiltration and fibrosis of lung tissue were observed under a Slide scanning system (#SQS-40R, Shengqiang Technology, Shenzhen, China).

### 4.6. Bioinformatics Analysis

The relationship among S1PR2, RHOA, YAP, Drp1, and MFN was evaluated with the String database.

### 4.7. Immunohistochemistry

The lung tissue sections were routinely deparaffinized and repaired with citrate antigen. After eliminating the endogenous peroxidase and blocking, the sections were sequentially incubated with primary antibodies (dilution ratio 1:200 to 500) overnight at 4 °C and the secondary antibodies. DAB was used for color development and hematoxylin was used for counterstaining. Finally, the sections were observed under a microscope.

### 4.8. TUNEL Assay

The lung tissue sections were routinely dehydrated and then incubated with 20 μg/mL DNase free proteinase K at 37 °C for 30 min, followed by incubation with 50 μL of TUNEL assay solution from Dead End Fluorometric TUNEL System at 37 °C for 60 min in the dark. Then, samples were analyzed using Cystation 5 (BioTek, Inc., Winooski, VT, USA).

### 4.9. Cell Culture and Treatment

Type 2 alveolar epithelial cells (MLE-12) cells were obtained from (Fuheng Biotechnology Co, Ltd., Shanghai, China). They were cultured in DMEM/F12 supplemented with 1% penicillin/streptomycin and 10% fetal bovine serum. To silence the expression of S1PR2 and YAP, MLE-12 cells were transfected with S1PR2 siRNA and YAP siRNA using Lipofectamine RNAiMAX for 24 h. Before induction with S1P (3 μmol/L), cells were pretreated with JTE-013 (2 μmol/L) for 2 h and then with Verteporfin (3 μmol/L) for 1 h. Cells were divided into the Control group, S1P group, siS1PR2 (JTE-013) group, S1P + siS1PR2 (S1P + JTE-013) group, and S1P + siS1PR2 + siYAP (S1P + JTE-013 + Verteporfin) group.

### 4.10. Western Blot

Total proteins were isolated from homogenized lung tissues and MLE-12 cells after lysis with RIPA and total protein concentrations were quantified by BCA assay kit. Western blot analysis detected the expression of RHOA/YAP pathway proteins, fibrosis marker proteins, and mitochondrial fusion/fission proteins. Protein levels were analyzed by Image J version 1.8 (National Institutes of Health, Bethesda, MD, USA).

### 4.11. MTT Assay

After the cells were starved in serum-free medium for 24 h, they were treated with S1P (1, 2, 3, 4, and 5 μmol/L) for 12, 24, and 48 h, respectively. The survival rate of the cells was detected by MTT assay, and the absorbance at 490 nm was measured on a full wavelength microplate detector.

### 4.12. Immunofluorescence Staining

Immunofluorescence staining was performed on lung tissues and MLE-12 cells. Briefly, tissue and cell samples were incubated with primary antibodies (1:200–500 dilutions) at 4 °C overnight. Then, incubation with fluorescent secondary antibodies (1:500 dilutions) for 2 h was conducted. Finally, DAPI was used to stain the nuclei. All samples were photographed by Cytaion 5 (BioTek, Inc., Winooski, VT, USA).

### 4.13. Mitochondrial Fission Analysis and Mitochondrial Membrane Potential Assay 

Cells were plated in 24-well glass plates and treated as above described. To assess mitochondrial fission, cells were treated with 400 nmol/L MitoTracker Red for 30 min at 37 °C. After that, cells were incubated with anti-Drp1 antibody overnight at 4 °C, followed by incubation for 2 h with fluorescent secondary antibody. To detect mitochondrial membrane potential, the treated cells were incubated with 5 μmol/L JC-1 staining solution at 37 °C for 20 min. Finally, samples were photographed by Cytation 5 (BioTek, Inc., Winooski, VT, USA).

### 4.14. Total ROS and Mitochondrial ROS Detection 

The ROS-specific fluorescent probe DCFH-DA (10 μmol/L) was added and stained for 10 min. To detect mitochondrial ROS, cells were stained with 5 μmol/L MitoSOX for 30 min at 37 °C. Finally, photographs were taken by Cytation 5 (BioTek, Inc., Winooski, VT, USA).

### 4.15. Flow Cytometry Analysis

IL-4 and TNF-α positive cells in BALF and spleen were detected by flow cytometry. Briefly, individual cells in BALF and spleen were first surface stained. Then, intracellular staining was performed with antibodies against IL-4 and TNF-α. Apoptosis of MLE-12 cells was analyzed with FITC Annexin V Apoptosis Detection Kit. All analyses were performed on the CytoFLEX flow cytometer (Beckman Coulter, Inc., Brea, CA, USA).

### 4.16. Statistical Analyses

Statistical analysis of all data was performed using GraphPad Prism version 8.0.2. Data of normal distribution are described by mean ± SD. A one-way analysis of variance (ANOVA) was conducted for the multi-group comparison, followed by Tukey’s post hoc test to evaluate the subsequent differences. *p* < 0.05 was considered statistically significant.

## 5. Conclusions

**Figure 7:**
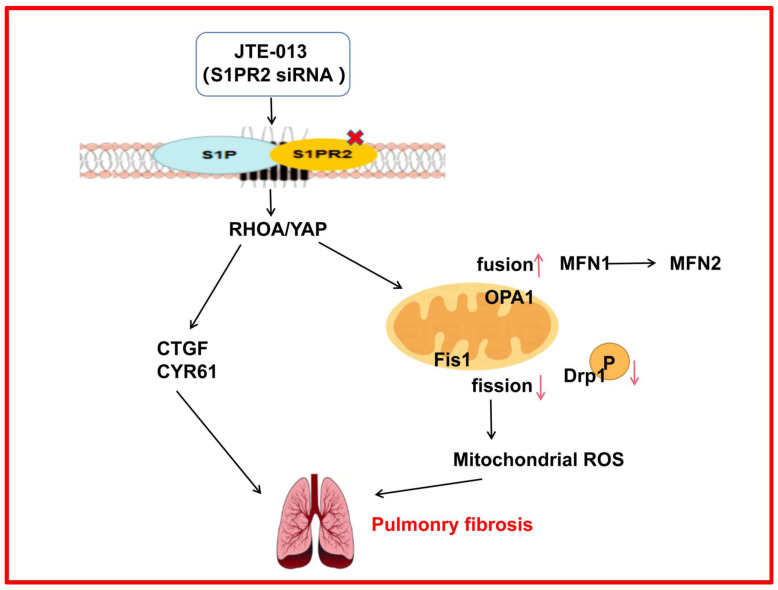
Schematic representation of the role of JTE-013 in pulmonary fibrosis. JTE-013 or S1PR2 knockdown inhibited the binding of S1P and S1PR2 to regulate the RHOA/YAP pathway. This further affected the downstream CTGF/CYR61 expression and mitochondrial dynamics, promoted mitochondrial fusion and the phenotypic transition of MFN, inhibited mitochondrial fission, down-regulated the phosphorylation level of Fis1 and Drp1, and reduced the production of mitochondrial ROS and ROS, thereby alleviating pulmonary fibrosis.

In summary, we found that JTE-013 could regulate mitochondrial function by inhibiting the expression of RHOA/YAP signaling pathway proteins, mediate mitochondrial fusion/fission signaling, and regulate ROS production, thus attenuating the development of pulmonary fibrosis (Figure 7). Therefore, JTE-013 exhibits significant potential in reducing pulmonary fibrosis by modulating the RHOA/YAP pathways and mitochondrial fusion/fission. However, the relationship between RHOA/YAP pathways and mitochondrial fusion/fission remains undetermined, which is a limitation of this study. Further studies are warranted. Collectively, our research provides valuable insights into the clinical diagnosis and management of pulmonary fibrosis. Furthermore, our findings suggest that JTE-013 holds promise as a potential therapeutic agent for treating pulmonary fibrosis.

## Data Availability

The datasets used and/or analyzed during the current study are available from the corresponding author upon reasonable request.

## Supplementary Materials

The following supporting information can be downloaded at: https://www.mdpi.com/article/10.3390/ph16101444/s1, Original Western blots.
