# Supplementary material for: JTE-013 Alleviates Pulmonary Fibrosis by Affecting the RhoA/YAP Pathway and Mitochondrial Fusion/Fission
Source: Pharmaceuticals (Basel). 2023 Oct 12;16(10):1444. doi: 10.3390/ph16101444 (PMC10609863; doi:10.3390/ph16101444)

Original Western blots for Figure 2

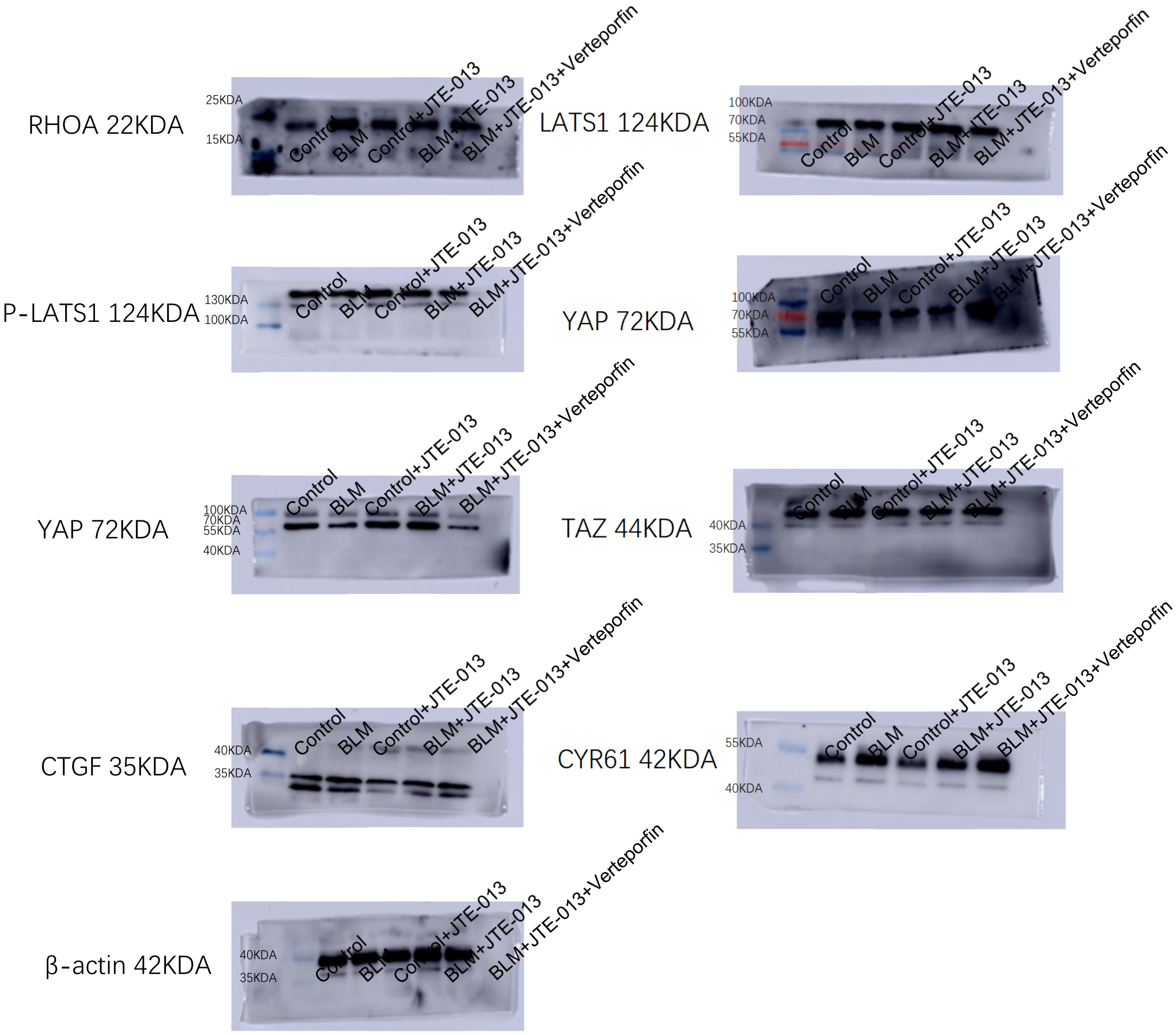

Original Western blots for Figure 3

DrP1 83KDA

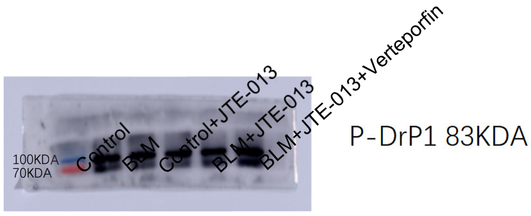

P-DrP1 83KDA

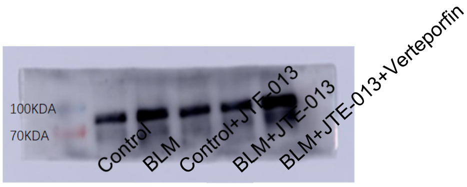

Fis1 17KDA

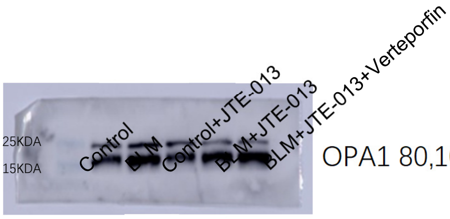

OPA1 80,100KDA

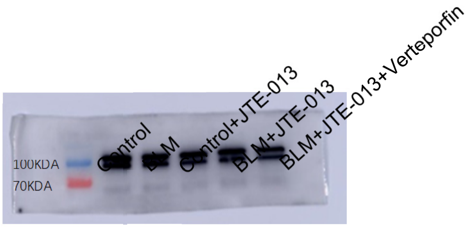

MFN1 86KDA

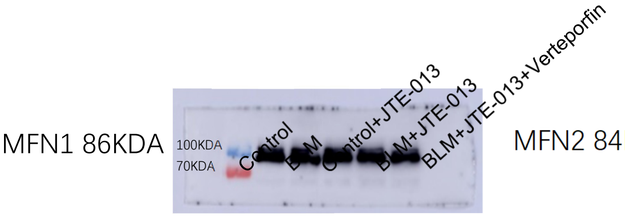

MFN2 84KDA

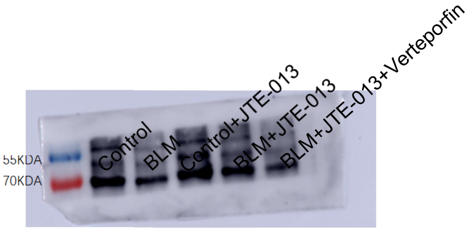

β-actin 42KDA

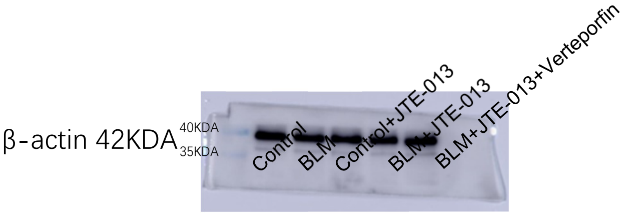

COL1A1 130KDA

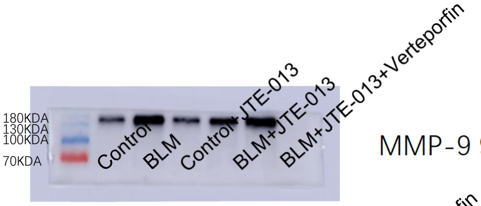

MMP-9 90KDA

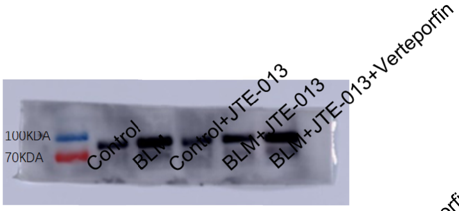

α-SMA 42KDA

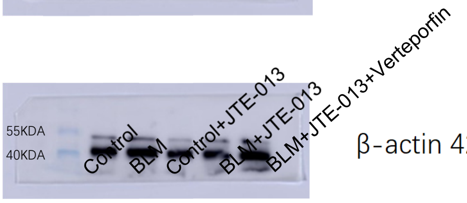

β-actin 42KDA

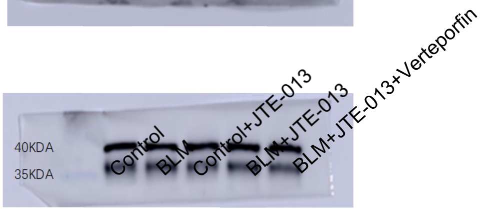

Original Western blots for Figure 4

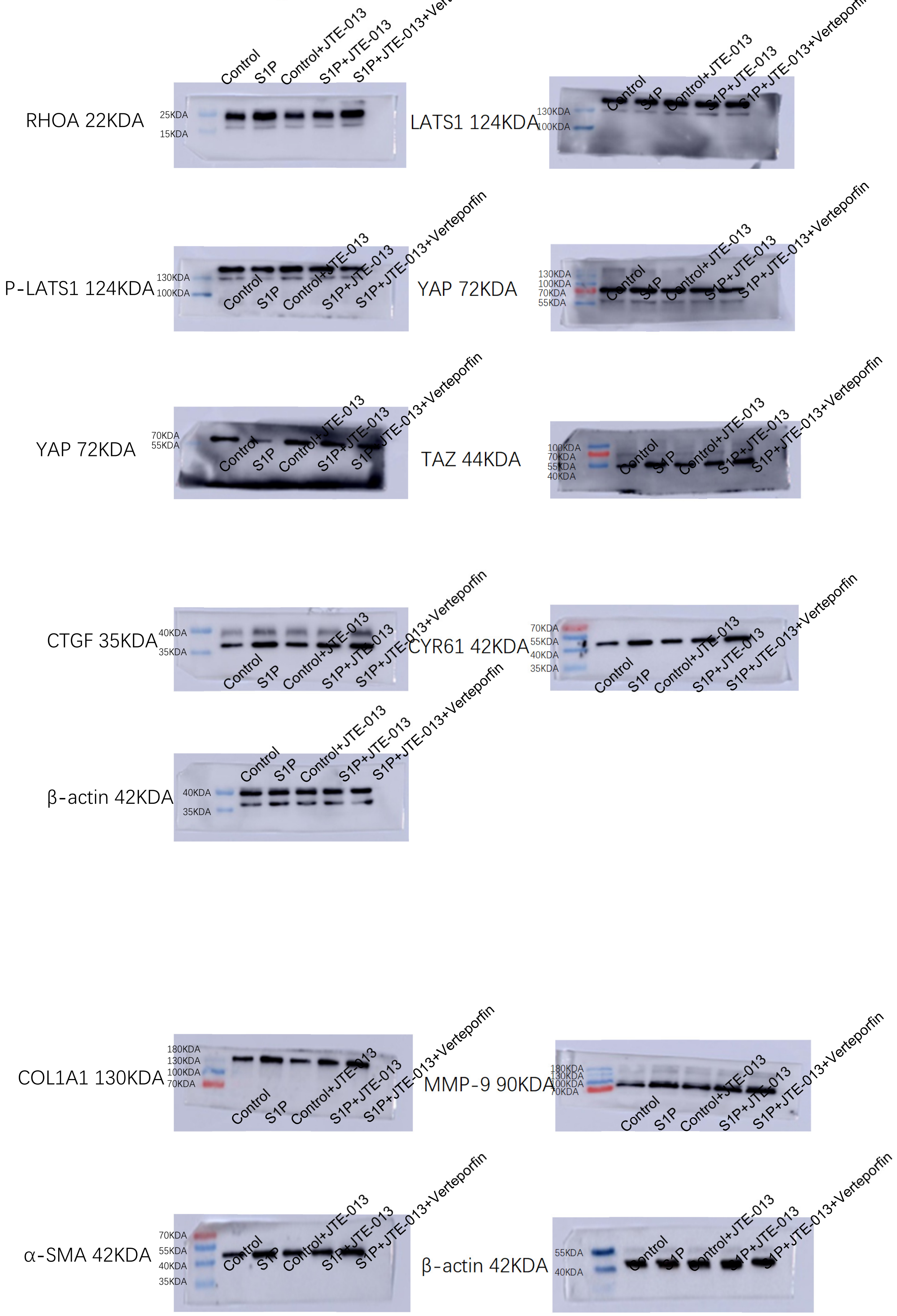

Original Western blots for Figure 5

DrP1 83KDA

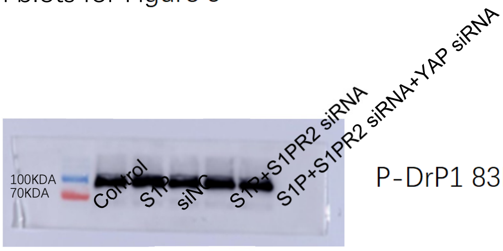

P-DrP1 83KDA

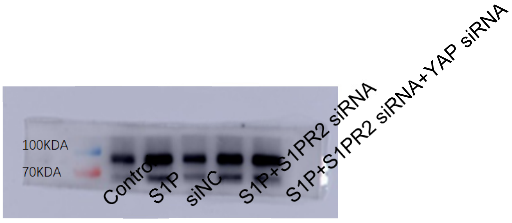

Fis1 17KDA

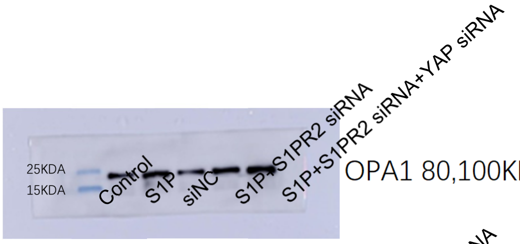

OPA1 80,100KDA

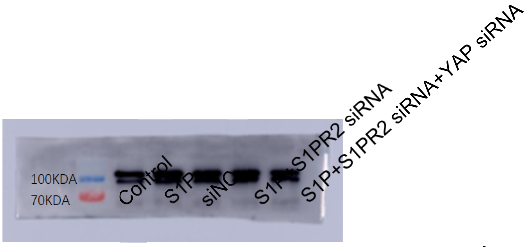

MFN1 86KDA

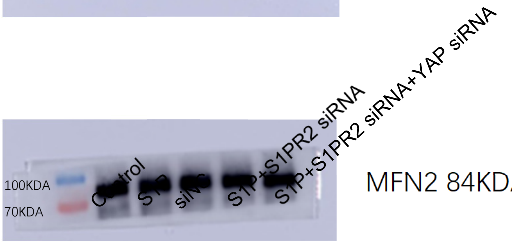

MFN2 84KDA

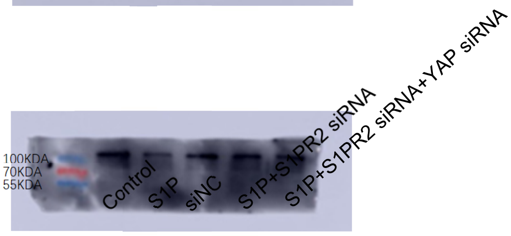

β-actin 42KDA

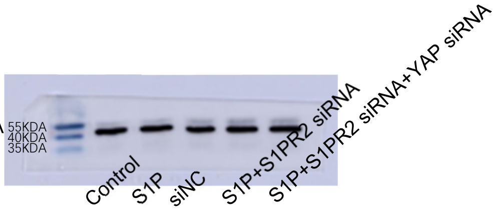

Original Western blots for Figure 6

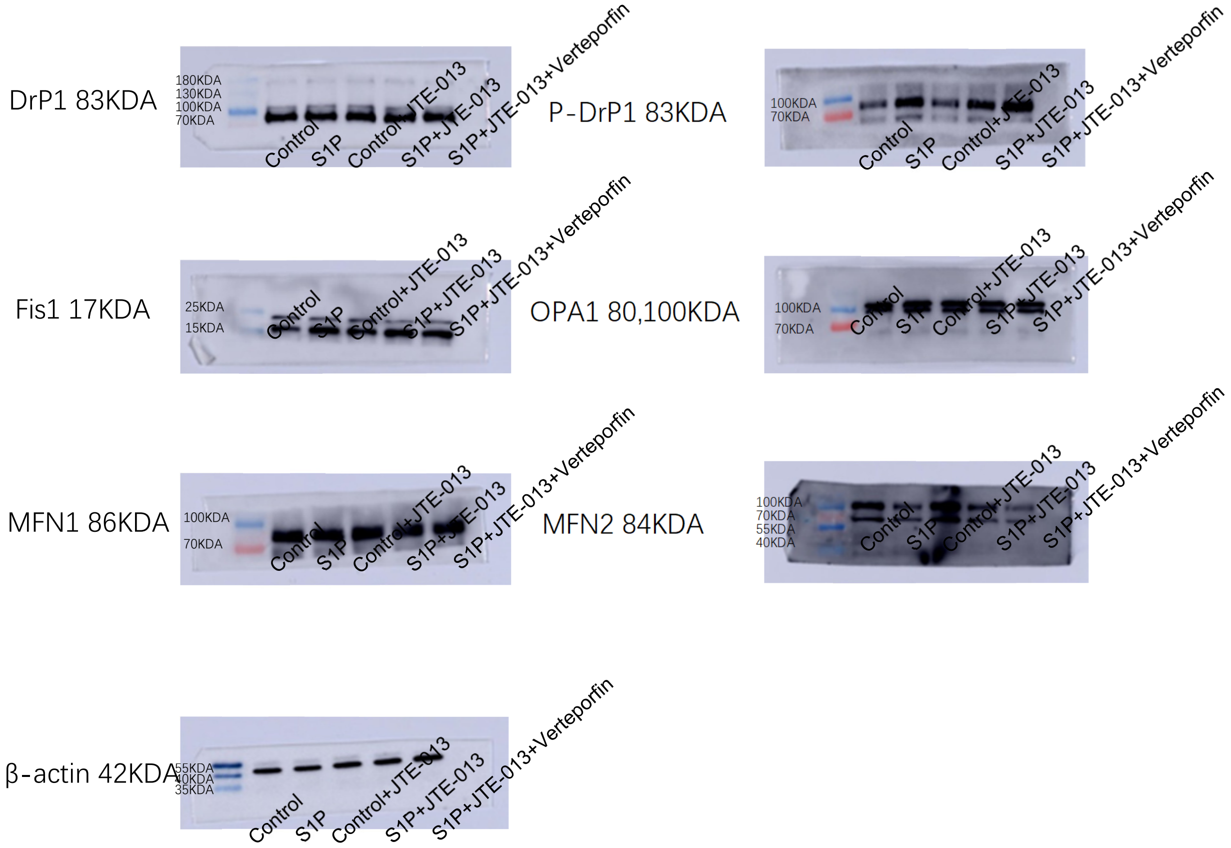

Supplement: Supplementary file 1 [file pharmaceuticals-16-01444-s001.zip › pharmaceuticals-2547968-supplementary.pdf]
